# Age group DNA methylation differences in lemon sharks (*Negaprion brevirostris*): Implications for future age estimation tools

**DOI:** 10.1002/ece3.9226

**Published:** 2022-08-29

**Authors:** Andria Paige Beal, Serena Hackerott, Kevin Feldheim, Samuel H. Gruber, Jose M. Eirin‐Lopez

**Affiliations:** ^1^ Environmental Epigenetics Laboratory, Institute of Environment Florida International University Miami Florida USA; ^2^ Pritzker Laboratory for Molecular Systematics and Evolution Field Museum of Natural History Chicago Illinois USA; ^3^ Bimini Biological Field Station Foundation South Bimini Bahamas

**Keywords:** age estimation, conservation, DNA methylation, MSAP, sharks

## Abstract

Age information is often non‐existent for most shark populations due to a lack of measurable physiological and morphological traits that can be used to estimate age. Recently, epigenetic clocks have been found to accurately estimate age for mammals, birds, and fish. However, since these clocks rely, among other things, on the availability of reference genomes, their application is hampered in non‐traditional model organisms lacking such molecular resources. The technique known as Methyl‐Sensitive Amplified Polymorphism (MSAP) has emerged as a valid alternative for studying DNA methylation biomarkers when reference genome information is missing, and large numbers of samples need to be processed. Accordingly, the MSAP technique was used in the present study to characterize global DNA methylation patterns in lemon sharks from three different age groups (juveniles, subadults, and adults). The obtained results reveal that, while MSAP analyses lack enough resolution as a standalone approach to infer age in these organisms, the global DNA methylation patterns observed using this technique displayed significant differences between age groups. Overall, these results confer that DNA methylation does change with age in sharks like what has been seen for other vertebrates and that MSAP could be useful as part of an epigenetics pipeline to infer the broad range of ages found in large samples sizes.

## Graphical abstract

Epigenetic clocks have been found to accurately estimate age for several species, however, these clocks rely heavily on the availability of reference genomes. This study explores the potential for using MSAP as an alternative to higher throughput methods for the analysis of age in sharks. Our results indicate that MSAP could be useful as part of an epigenetics pipeline to infer broad age ranges.

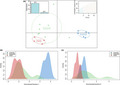

## 1 INTRODUCTION

Overfishing constitutes the most important threat to elasmobranchs including sharks, skates, and rays (Dulvy et al., 2014), making fisheries management critical for their conservation. Consequently, the characterization of demographic information is of utmost importance to model the sustainability of populations under fishing pressure. Age information is a critical parameter of these models, which is unfortunately not available for most species due (among other reasons) to the lack of physiological and morphological traits that can be accurately used as proxies of chronological age in elasmobranchs. Particularly in the case of sharks, body length has been largely used to infer age and has often been cross‐validated with vertebral growth bands (Carlson & Goldman, 2007). Yet, recent studies have reported that the age of older sharks has been vastly underestimated given that vertebral bands do not necessarily form on a yearly basis throughout a shark's lifetime (Natanson et al., 2018; Natanson & Skomal, 2015). Not less importantly, vertebral band counts are highly invasive, require sacrificing animals, and do not work for all species (Francis et al., 2007; Huveneers et al., 2013; Natanson et al., 2018).

The emergence of methodological approaches linking molecular variation to chronological age has constituted a breakthrough, particularly in those cases where age estimation has proven difficult and/or based on invasive methods. The characterization of distinctive molecular traits is possible using small tissue samples that can be more easily obtained, in most cases without even the need to capture/release the animal. One of the first among these methods was based on the quantification of the length of telomeres, based on their progressive shortening during successive cell cycle divisions. While telomere‐based aging methods proved to be a great proxy for biological age (i.e., how old an animal looks for its age), the vast differences in the starting length observed in telomeres led numerous studies to question its applicability for determining chronological age estimation (i.e., actual age of the organism) (Nehmens et al., 2021; Remot et al., 2020; Vaiserman & Krasnienkov, 2020). Epigenetics, defined as “the study of molecules and mechanisms that can perpetuate alternative gene activity states in the context of the same DNA sequence” (Cavalli & Heard, 2019), has provided a basis to start solving that problem by establishing direct links between chronological age and particular epigenetic modifications including DNA methylation, histone variants, histone modifications, and small RNAs (López‐Otín et al., 2013), providing not only insights into how organisms age but also facilitating the estimation of age using DNA methylation (the addition of a methyl group most commonly to a cytosine in the DNA sequence) as a proxy (Horvath, 2013).

Over the last decade, several epigenetic clocks using DNA methylation to estimate chronological (actual or real age) and biological age (physiological age, how old an individual seems) have been successfully developed across diverse taxa (Beal et al., 2019; Bors et al., 2021; Chen et al., 2016; De Paoli‐Iseppi et al., 2019; Horvath & Raj, 2018; Polanowski et al., 2014; Salameh et al., 2020). Chronological epigenetic clocks specifically correlate percent DNA methylation at CpG sites (a Cytosine followed by a Guanine in the same DNA strand) to age through multiple regression analyses producing highly accurate estimations (correlation coefficient, *r* ^2^, ranging from 0.70 to 0.99). These changes in DNA methylation are linked to age‐related mechanisms and developmental milestones that often result in changes in gene expression (Gladyshev, 2016; Hannum et al., 2013; López‐Otín et al., 2013). Although largely focused on mammalian species (Barratclough et al., 2021; Beal et al., 2019; Bors et al., 2021; Fei et al., 2021; Horvath & Raj, 2018; Polanowski et al., 2014; Robeck et al., 2021; Stubbs et al., 2017; Tanabe et al., 2020), accurate clocks have been developed for other taxa including birds and fishes (Anastasiadi & Piferrer, 2020; De Paoli‐Iseppi et al., 2019; Mayne et al., 2020).

Chronological epigenetic clocks get their accuracy from the characterization of DNA methylation at particular DNA positions using high‐resolution approaches such as microarrays, reduced representation bisulfite sequencing, pyrosequencing, and qPCR (Morselli et al., 2021; Trigg et al., 2021). However, such techniques are hampered by two major limitations: the requirement of a sequenced reference genome for the species studied and the potentially high cost of conducting these analyses in large numbers of samples to be ecologically meaningful in field studies, particularly for the larger sample sizes necessary for ecologically relevant population and stock assessments. Thus, the application of less demanding and inexpensive coarse‐grained methods, still providing useful aging information at the level of age intervals, represents a compromise helping the characterization of aging structures in natural populations within realistic ecological settings. The characterization of Methyl‐Sensitive Amplified Polymorphism (MSAP) is one such method, based on the use of dual methylation‐sensitive restriction enzymes to unveil broad DNA methylation patterns found at target cut site 5’‐CCGG‐3′ (invented by Reyna‐López et al., 1997). This method has been successfully used to examine the links between environmental change and genome‐wide DNA methylation patterns involved in phenotypic responses (Beal et al., 2021; Huang et al., 2017; Morán et al., 2013; Pierron et al., 2014; Rodríguez‐Casariego et al., 2020; Suarez‐Ulloa et al., 2019; Zhao et al., 2015).

Since it is well known that gene expression patterns change during development and aging, the observation of global amounts of DNA methylation changing over an individual's lifetime comes as no surprise (Heyn et al., 2012). However, it is not yet known if such changes are large enough to be efficiently detected using MSAP as a standalone epigenetic aging method. The present study addresses that question by using MSAP in lemon shark (Negaprion brevirostris) samples belonging to three age groups (juvenile, subadult, and adult), available through the unique long‐term sampling archive from Bimini, Bahamas. This archive of samples is one of the most comprehensive datasets for sharks and is the best known age dataset to date for any shark species. The MSAP method used in the present study was previously adjusted for use in lemon sharks from populations monitored in our own research (Beal et al., 2021). The obtained results are consistent with the applicability of MSAP analyses as part of a larger epigenetic pipeline to determine chronological age, particular at earlier stages, to define age groups in large numbers of samples which can be subsequently more finely characterized using single nucleotide resolution methods.

## 2 METHODS

### 2.1 Samples and DNA extraction

Lemon sharks, as studied in Bimini (Bahamas) by the Bimini Shark Lab for more than 30 years, exhibit a life history of biennial parturition after reaching sexual maturity around age 12 (Brown & Gruber, 1988; Feldheim et al., 2004) as well as natal philopatry where they return back to the particular nursery site where they were born (Feldheim et al., 2014). Although age of reproductive senescence is unknown, pregnant females in the low to mid‐30s have been recorded in the Bimini area and the maximum lifespan is estimated at 37 years (Brooks et al., 2016).

Epigenetic age estimation has been successful across multiple tissue types (Horvath, 2013). Therefore, we focused on fin clips as this is the type of tissue sample most often collected during shark population monitoring efforts and therefore most relevant for managers and conservation practitioners. All lemon shark samples analyzed in this study consisted of fin clips housed in an archived collection at the Field Museum in Chicago with collection dates ranging from 1995 to 2017. Most samples were collected in Bimini, Bahamas (Bahamas Research Permits MA&MR/FIS/17B) except for three samples that were collected in Jupiter, FL (Table 1). All fin clips were preserved in Dimethyl Sulfoxide (DMSO) at room temperature for up to 2 years and then stored in −20°C freezer thereafter. Upon original collection, samples were designated as one of the following age groups/life stages following Feldheim et al. (2014). Newborn sharks had an open umbilical scar, and juvenile sharks had closed umbilical scars but did not exceed 70 cm precaudal length (PCL) (1–5 years). Subadults (6–11 years) were between 70–175 cm PCL for males and 70–185 cm for females, and adults (12+ years) were greater than 175 cm PCL for males and greater than 185 cm for females.

**TABLE 1:** Samples and metadata. Batch 1 samples include LS1‐LS10. Batch 2 includes all remaining samples listed

ID	Batch	Capture date	Age group for testing	Sex	Collection site
LS1	1	2/2/2017	Subadult	M	Bimini
LS2	1	12/12/2016	Adult	M	Bimini
LS3	1	3/16/2009	Subadult	F	Jupiter
LS4	1	2/22/2009	Adult	F	Jupiter
LS5	1	2/21/2009	Adult	F	Jupiter
LS6	1	1/29/2013	Juvenile	M	Bimini
LS7	1	7/16/2012	Juvenile	M	Bimini
LS8	1	5/31/2013	Juvenile	M	Bimini
LS9	1	6/12/2012	Subadult	M	Bimini
LS10	1	2/2/2015	Juvenile	F	Bimini
LS11	2	4/16/2004	Subadult	F	Bimini
LS12	2	3/25/2004	Subadult	F	Bimini
LS13	2	4/7/2004	Subadult	M	Bimini
LS14	2	11/24/1996	Subadult	M	Bimini
LS15	2	7/15/1997	Subadult	M	Bimini
LS16	2	7/16/1997	Subadult	F	Bimini
LS17	2	7/23/1997	Subadult	M	Bimini
LS20	2	3/9/1997	Subadult	M	Bimini
LS22	2	5/9/2008	Adult	F	Bimini
LS24	2	2/25/1996	Adult	F	Bimini
LS25	2	6/8/1996	Adult	M	Bimini
LS26	2	7/21/1996	Adult	F	Bimini
LS29	2	6/16/1994	Subadult	F	Bimini
A13	2	7/7/2009	Juvenile	F	Bimini
A14	2	7/8/2009	Juvenile	F	Bimini
A59	2	6/20/2002	Juvenile	M	Bimini
A60	2	6/20/2002	Juvenile	M	Bimini
A61	2	6/19/2002	Juvenile	F	Bimini
A62	2	6/20/2002	Juvenile	F	Bimini

The samples used in this study were processed in two batches. The first batch (Batch 1, LS1‐10) was analyzed as part of a proof‐of‐concept pilot study, where DNA extraction was performed at the Field Museum in Chicago using a salting‐out protocol (Sunnucks & Hales, 1996). These samples were preserved in ice and shipped to Florida International University (FIU) for MSAP analyses. Batch 2 samples were shipped as fin clip samples in DMSO, and DNA was subsequently extracted following the same salting‐out protocol used for Batch 1 samples (Beal et al., 2021). Batch 1 samples (*n* = 10) consisted of four juveniles, three subadults, and three adults. Batch 2 samples (*n* = 19) consisted of six juveniles, nine subadults, and four adults.

### 2.2 DNA methylation pattern analysis

DNA methylation patterns were determined using MSAP analyses. This method employs dual methylation‐sensitive enzymes that cut differentially at the same target site 5’‐CCGG‐3′ (Reyna‐López et al., 1997) creating dual fragment profiles identifying four types of DNA methylation states present (full or hypermethylation, internal C methylation, hemi‐methylation, or no methylation). MSAP analyses were conducted independently for each of the two batches of samples as detailed in Beal et al. (2021). Briefly, a parallel restriction digestion/ligation (each containing one of the methyl‐sensitive restriction enzymes MSPI or HPAII and each containing the non‐methyl sensitive rare cutting restriction enzyme EcoRI) was performed using 200 ng of DNA, followed by a pre‐selective PCR targeting the ligated ends with an additional base pair, and ending with a final selective PCR with an additional two base pairs added to the primer along with a fluorescent dye (6‐FAM) used to detect fragment length (Table 2). A total of four selective primers in two combinations were used for both batches. Fragment analysis was conducted by the FIU DNA Core on an Applied Biosystems® 3130XL Genetic Analyzer. Raw fragment data were then scored as presence (+)/ absence (−) for all fragments for each individual for each parallel reaction (MSPI and HPAII) to decipher the four types of DNA methylation status present. Presence (+)/absence (−) (MSPI/HPAII) pattern for each fragment gave methylation type (+/+ = unmethylated, +/− = hemi‐methylated, −/+ = internal C methylation, −/− = full methylation). Batch 1 samples were subjected to the MSAP protocol and fragment analysis at a separate and earlier time than Batch 2 samples. Once raw data were available for all samples, the final dataset was scored using all samples to allow for loci to be compared.

**TABLE 2:** Adaptors and Primers usedfor MSAP analyses. Only selective primers were fluorescently labeled (FAM) for fragment analysis

Step/combo	Oligo name	Sequence (5′ to 3′)
Restriction ligation	EcoRI Adaptor Fwd	CTCGTAGACTGCGTACC
Restriction ligation	EcoRI Adaptor Rv	AATTGGTACGCAGTCTAC
Restriction ligation	HPAII/MSPI Adaptor Fwd	CGTTCTAGACTCATC
Restriction ligation	HPAII/MSPI Adaptor Rv	GACGATGAGTCTAGAA
Preselective combo A	Pre‐EcoRI + A	GACTGCGTACCAATTCA
Preselective combo A	Pre‐MspI‐HpaII + T	GATGAGTCTAGAACGGT
Preselective combo B	Pre‐EcoRI + C	GACTGCGTACCAATTCC
Preselective combo B	Pre‐MspI‐HpaII + A	GATGAGTCTAGAACGGA
Selective Combo 1	selective MspI‐HpaII‐TTG	GATGAGTCTAGAACGGTTG‐FAM
Selective Combo 1	selective MspI‐HpaII‐TCT	GATGAGTCTAGAACGGTCT‐FAM
Selective Combo 2	selective EcoRI‐AAC	GACTGCGTACCAATTCAAC‐FAM
Selective Combo 2	selective MspI‐HpaII‐TCA	GATGAGTCTAGAACGGTCA‐FAM

### 2.3 Data analysis

The *msap* package (version 1.1.8) in R was used to categorize each locus as either unmethylated, hemi‐methylated, methylated at an internal cytosine, or fully methylated, for each sample (Pérez‐Figueroa, 2013). Although a lack of bands in both reactions could indicate full methylation or the lack of a genetic target, this case was considered as full methylation, consistent with other DNA methylation studies working with species where there is low genetic structure (Feldheim et al., 2001; Rodríguez‐Casariego et al., 2020), as has been shown in lemon sharks (Feldheim et al., 2001). Methylation‐susceptible loci (MSL) were identified as loci with a methylated state in at least 5% of the samples (Pérez‐Figueroa, 2013), and polymorphic loci were identified as those that displayed both methylation and a lack of methylation in at least two instances across samples (Herrera & Bazaga, 2010; Pérez‐Figueroa, 2013). Polymorphic MSL were the focus of all subsequent analyses of DNA methylation. For all distance‐based analyses, Gower distances based on the four possible methylation states (u, h, I, or f) were utilized (*daisy* function, *cluster* package, version 2.1.0; Gower, 1971).

Initially, all samples were analyzed together. A Non‐metric Multidimensional Scaling Analysis (NMDS; *metaMDS* function, *vegan* package version 2.5–6; Faith et al., 1987) was used to visualize patterns of DNA methylation and potential influencing factors including age group, year, and season of sampling, along with sample batch. Given the potential influence of differences in sample processing between batches, each batch was then analyzed separately. Each batch was re‐analyzed individually with the *msap* package to characterize methylation states and to identify polymorphic MSL within each batch. Differences in DNA methylation patterns between age groups in each batch were visualized with NMDS analyses and evaluated with permutational analysis of variance (PERMANOVA; *adonis* function; Anderson, 2001). The homogeneity of dispersions between age groups of each batch was assessed with the *betadisper* function (Anderson et al., 2006).

Given the larger sample size of Batch 2, differences in DNA methylation patterns between age groups were further assessed within this batch using a Discriminant Analysis of Principal Component (DAPC; *adegenet* package version 2.1.3; Jombart & Ahmed, 2011). The optimal number of principal components (PCs) to be retained (4 PCs) was determined through the *xval‐Dapc* function as well as an assessment of a‐scores (*optim.a.score* function; Jombart et al., 2010). Both discriminant functions were retained. To assess the separation between age groups, the probability of membership of each sample to each age group was evaluated. A distance‐based redundancy analysis (dbRDA; Legendre & Anderson, 1999; Oksanen et al., 2019) was performed with batch 2 samples to characterize the relationship between all the metadata available for the samples (age class, sex, year, season) and DNA methylation variation. The equation was as follows:

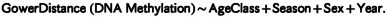




A permutation test was performed on the dbRDA model (anova.cca function) to assess the significance of these variables upon the model (Legendre et al., 2011).

## 3 RESULTS

### 3.1 Combined batch sample analysis

**FIGURE 1:**
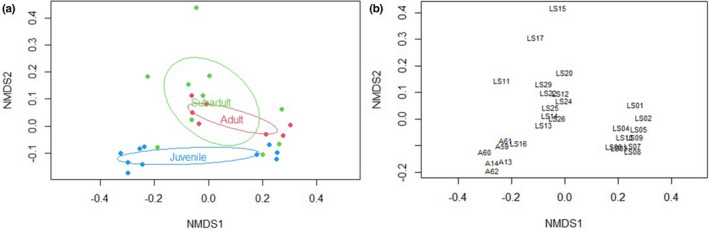
NMDS for Combined Samples (Batch 1 & 2). Both NMDS plots relay how similar/different each point is to another based on DNA methylation pattern. Plot A shows age groups for all samples. Plot B shows individual sample IDs where batch 1 (LS1‐10) clearly clusters together (towards right) and away from batch 2 samples (all other samples).

A preliminary MSAP analysis using Batch 1 samples (*n* = 10) revealed significant differences between age groups (Analysis of molecular variance (AMOVA), *p* < .05, Figures S1 and S2). In order to validate these findings, additional samples were incorporated into the analyses (Batch 1 + Batch 2 samples). The combined batch analysis encompassed a total of *n* = 29 samples (10 juveniles, 12 subadults, and seven adults) used in R analyses, specifically the *msap* package (Table 1). Two selective primer pairs (Table 2) were used which resulted in a total of 364 loci (133 and 231 for primers 1 and 2, respectively), out of which 346 were identified as MSL of which 76% were polymorphic and 18 as non‐methylated loci (NML) of which 33% were polymorphic using the *msap* package (additional information from MSAP and other R analyses can be found in the supplemental R Code file). AMOVA analyses indicated significant differences between age groups for MSL (Phi_ST = 0.108, df = 28, *p* = .003) but not for NML (Phi_ST = 0.005, df = 28, *p* = .387). The NMDS plot showed juveniles to overall cluster toward the bottom of the plot and adults toward the top with subadults having a large amount of variation; however, there was an observable difference in clustering between samples from Batch 1 and Batch 2 (Figure 1). The potential effect of differences in year of sampling and season was investigated, but neither appeared to have a significant influence in the clustering of data points (Figures S3 and S4). The only obvious difference appeared to be the fact that the samples were processed in two separate batches that were not processed by the same lab for all steps. Therefore, it was decided to investigate age group clustering within each batch effort to eliminate interference from the different sample processing and storage variables. Despite this apparent batch effect, age group differences were readily obvious and appeared robust despite possible differences between batches.

### 3.2 Independent batch sample analysis

**FIGURE 2:**
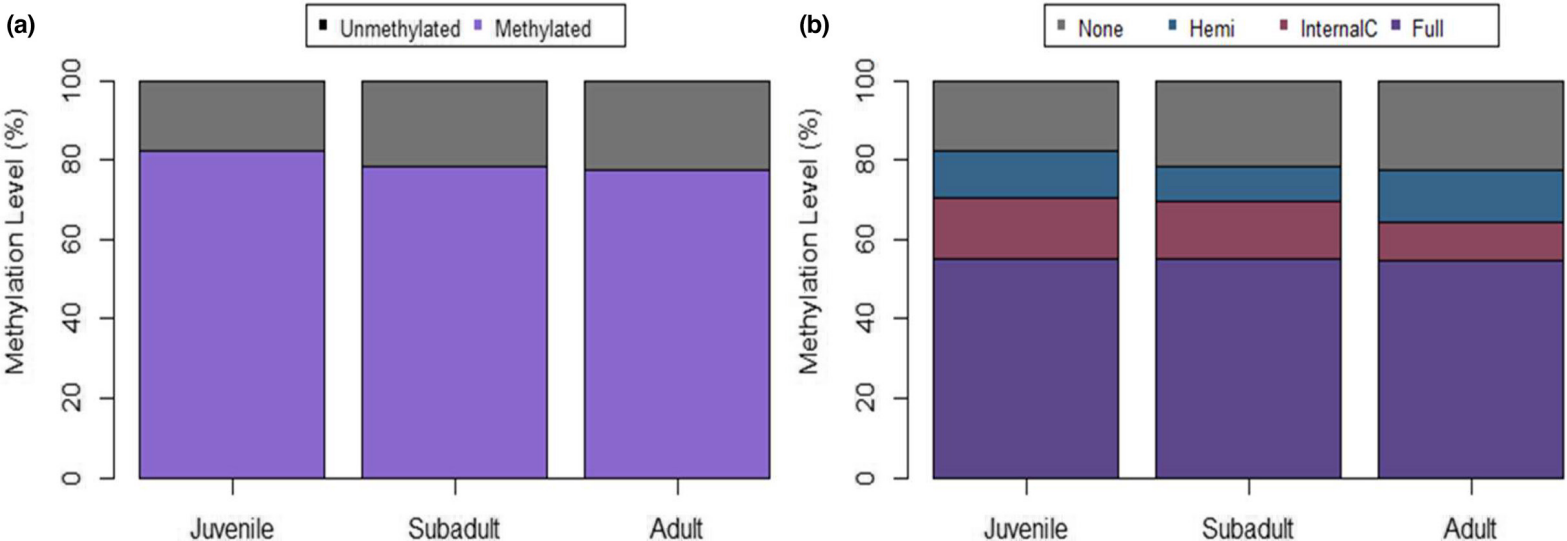
Percent DNA methylation per age Group for Batch 1 samples. Plot (a) depicts % loci as methylated or non‐methylated. Plot (b) depicts % loci for each of the four types of methylation status determined by MSAP analysis (none‐ non‐methylated, hemi‐hemi‐methylated, Internal C‐ internal cytosine methylated, full‐full/hypermethylated target site).

**FIGURE 3:**
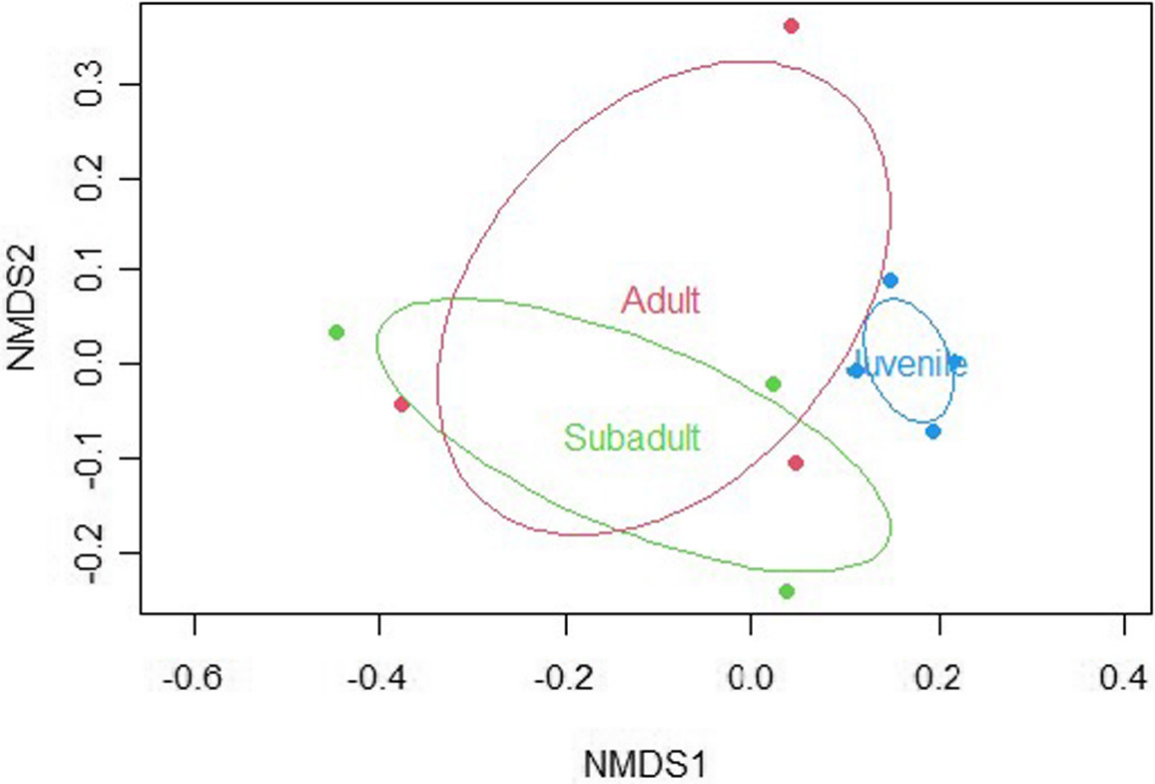
NMDS plot for batch 1. Distance between any two dots depicts the difference/similarity between two given samples

The analysis of Batch 1 samples (*n* = 10) consisted of four juveniles, three subadults, and three adults. A total of 308 loci were identified as MSL (45% of them polymorphic) and 56 to as NML (0% polymorphic) using the msap package (Figures S5 and S6). AMOVA analyses revealed a significant difference between age groups for MSL (Phi_ST = 0.111, df = 9, *p* = .049). Accordingly, juveniles had less unmethylated loci compared to subadult and adult groups (Figure 2). The NMDS plot showed that juveniles cluster more closely together than individuals in subadult or adult groups (Figure 3). Similarly, PERMANOVA analyses found a significant difference between age groups (*p* = .05) and a marginally significant difference in dispersion between groups (*p* = .09). Such age group differences were similar to those observed in the combined batch sample analysis, with samples from juvenile sharks showing a consistent clustering separate from other age groups, and with subadults displaying the most variation among data points.

**FIGURE 4:**
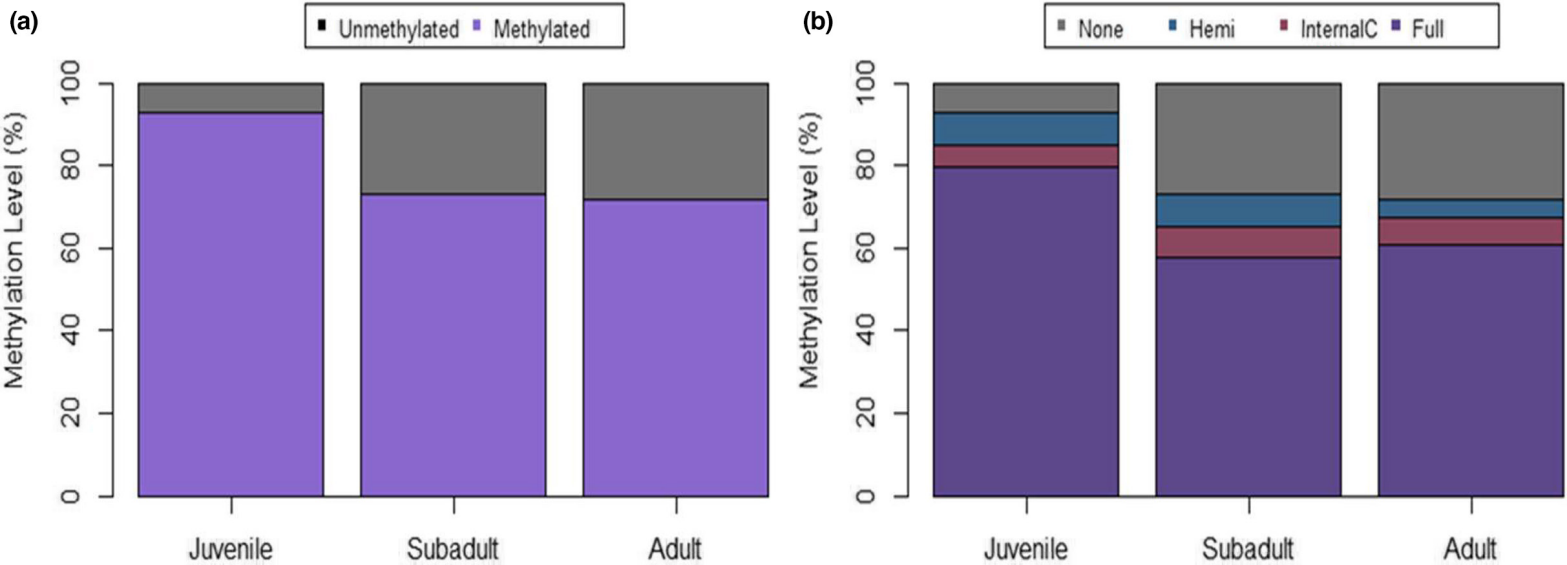
Percent DNA methylation per age Group for Batch 2. Plot (a) depicts % loci as methylated or non‐methylated. Plot (b) depicts % loci for each of the four types of methylation status determined by MSAP analysis (none‐non‐methylated, hemi‐hemi‐methylated, Internal C‐ internal cytosine methylated, full‐full/hypermethylated target site).

**FIGURE 5:**
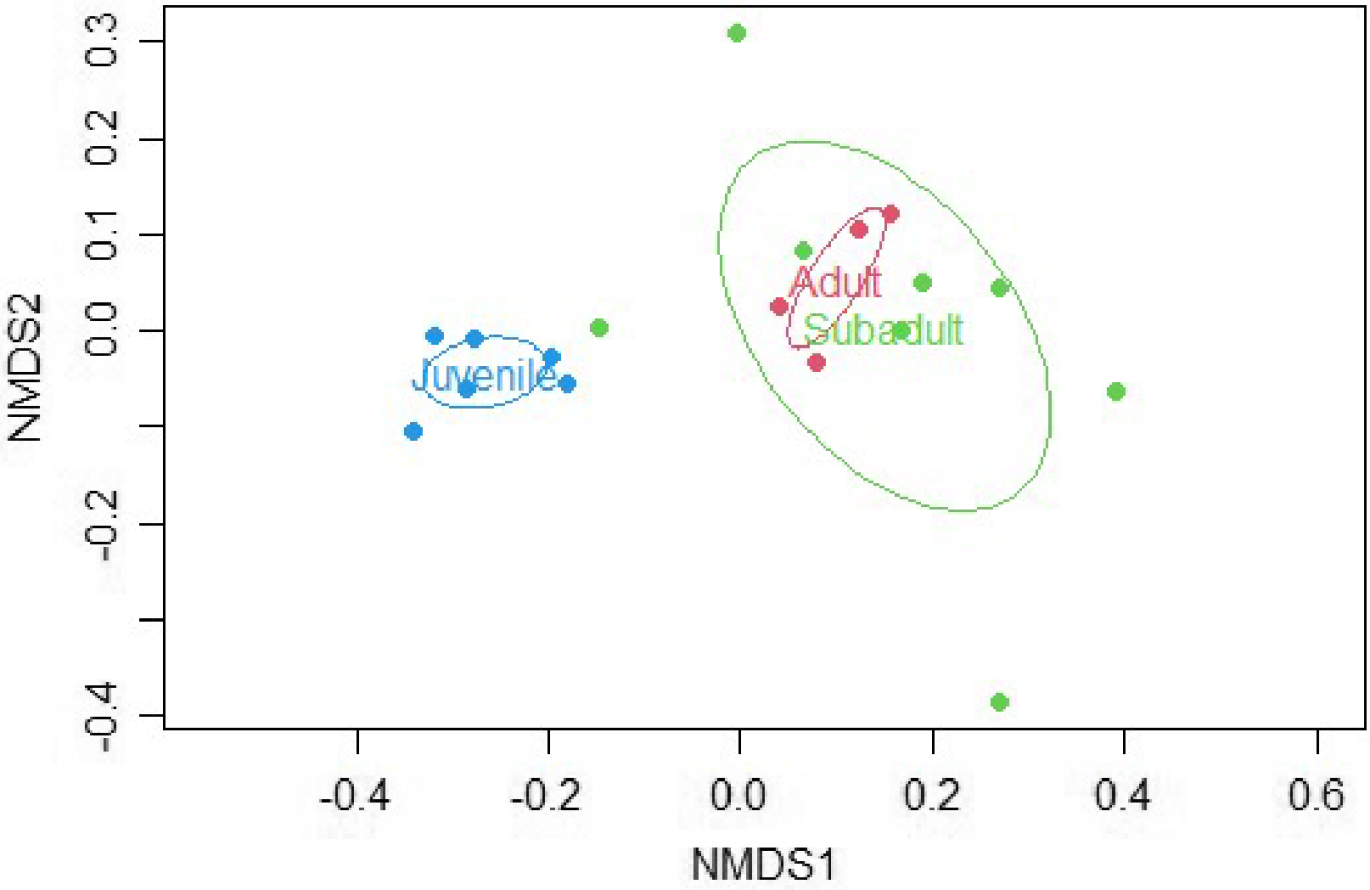
NMDS plot for batch 2. Distance between any two dots depicts the difference/similarity between two given samples

**FIGURE 6:**
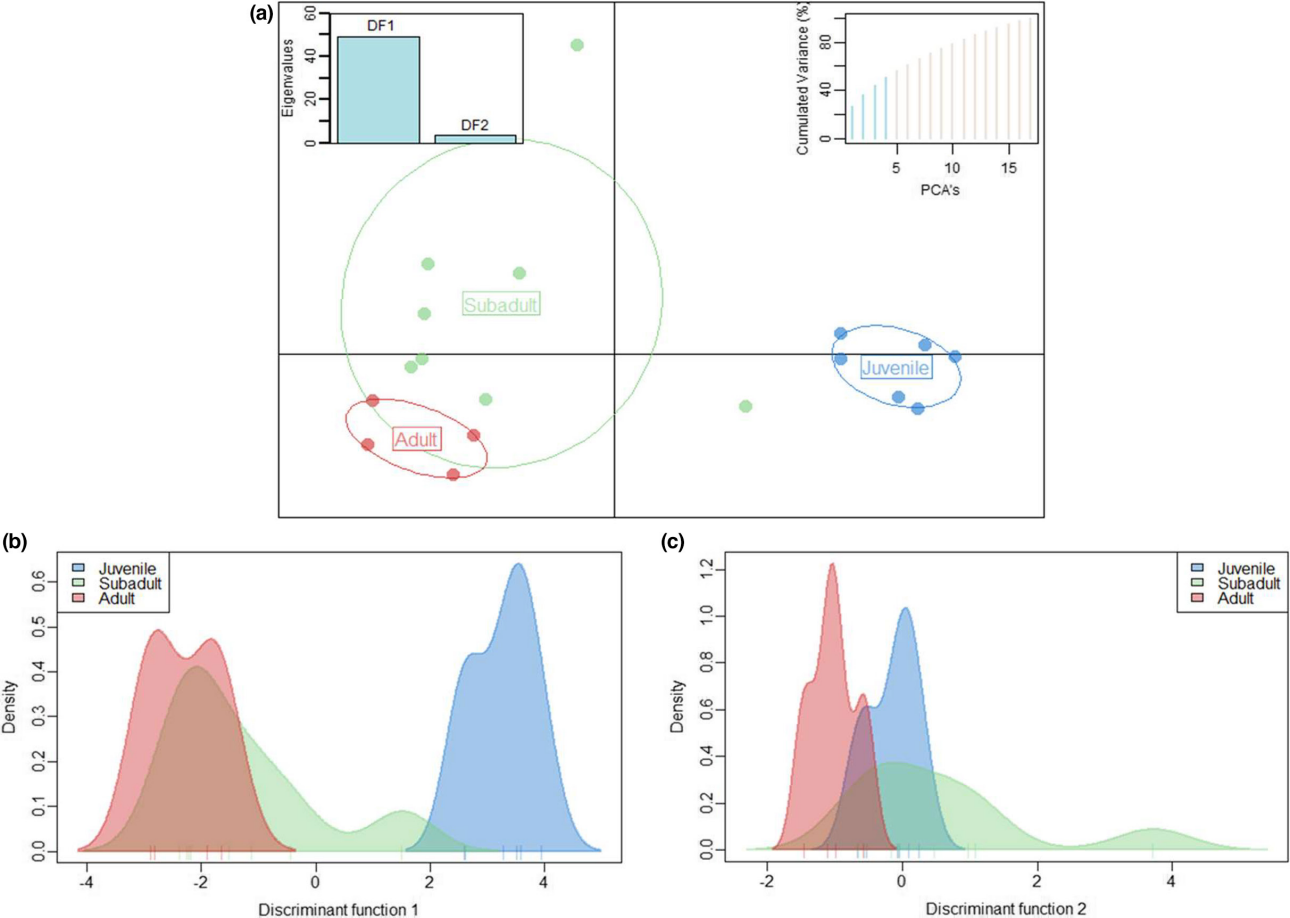
DAPC and density plots for discriminant function 1 and 2 for batch 2. Plot (a), shows spatial and temporal distribution of data points for the three age groups, *x*‐axis is DF1 and *y*‐axis is DF2. Plot (b), density plot for DF1 showing the most differentiation of juveniles from other age groups. Plot (c), density plot for DF2. In both B and C plots, we see that subadults have the most variation across both DF1 and DF2.

**FIGURE 7:**
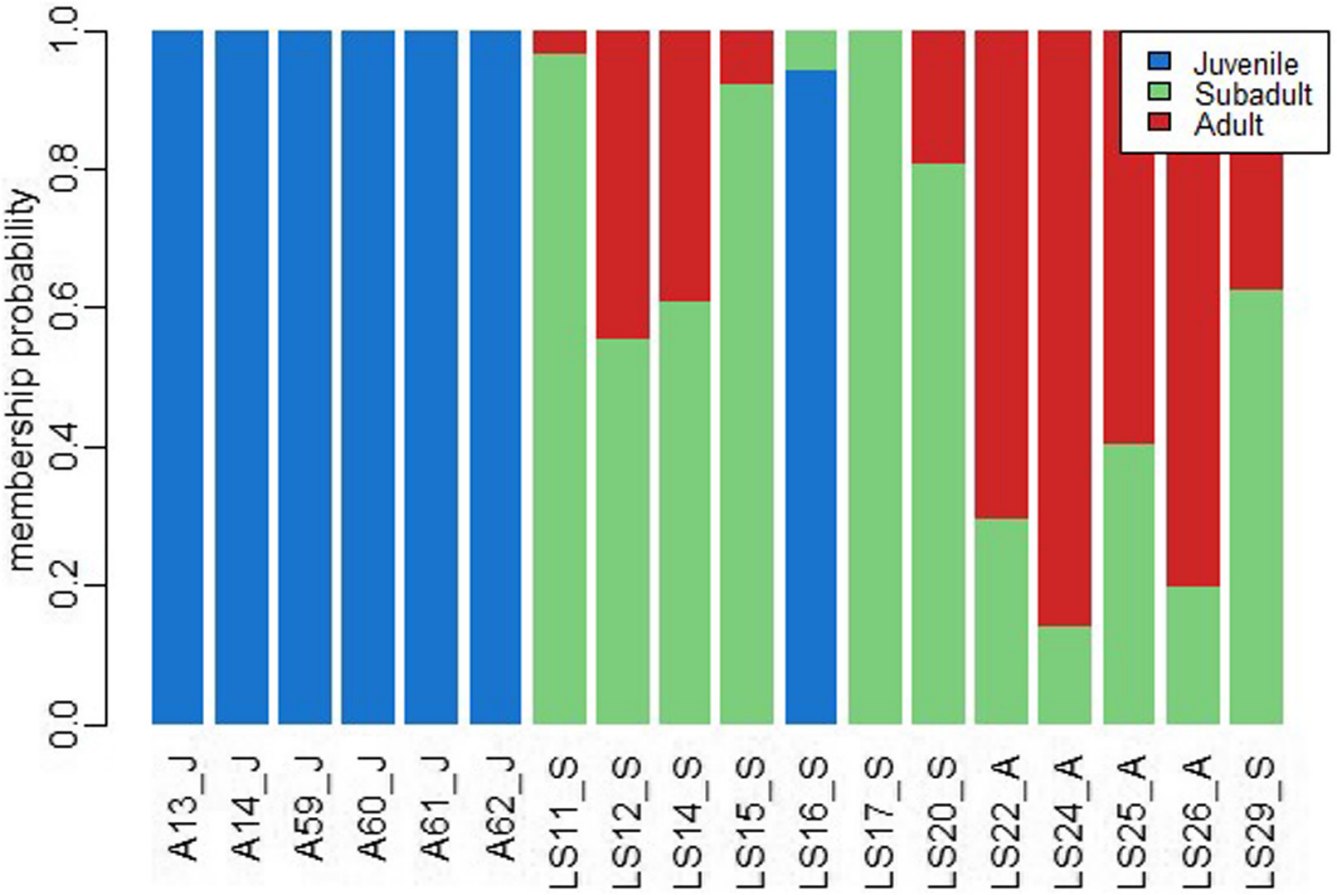
Probability of membership to a given age group. Samples are listed on the *x*‐axis as sample ID_Letter representative of age group as follows: J‐juveniles, S‐subadult, and a‐adult. Juveniles showed a 100% probability of being assigned to their correct group. Subadults had the most variation (50%–95%) in probability of correct assignment, whereas all adult samples had 60% or greater probability.

The analysis of Batch 2 samples consisted of *n* = 19 samples, including six juveniles, nine subadults, and four adults. This analysis revealed the presence of 341 MSL (62% polymorphic) and 23 NML (26% polymorphic). MSAP Package PCoA graph and NMDS plot for Batch 2 samples are in Figures S7 and S8. The AMOVA analysis of MSL data found a significant difference between age groups (Phi_ST = 0.308, df = 18, *p* < .0001), whereas AMOVA of NML did not (Phi_ST = 0.023, df = 18, *p* = .311). Again, juveniles displayed less unmethylated loci compared to subadults and adult groups, and a large portion of loci was found to be fully methylated in juveniles for this batch effort (Figure 4). Similar to the combined analysis and Batch 1 analysis, the NMDS plot for batch 2 showed samples from juvenile sharks clustering independently from subadults and adults (Figure 5). PERMANOVA analysis identified significant differences between age groups and dispersion between age groups (*p* = .001 and *p* = .007, respectively). To further analyze differences between age groups, a DAPC analysis was conducted on Batch 2 samples (feasible in this particular case based on its larger sample size) with discriminant function 1 accounting for the majority (~50%) of differentiation observed between juveniles and other age groups (Figure 6). Lemon shark age groups (i.e., juvenile, subadult, and adult) showed differential DNA methylation patterns (Figures 5 and 6), with the juvenile age group showing the most differentiated pattern from the other groups and with 100% accurate membership predictability compared with individuals from the other two age groups (Figure 7). These results are consistent with a progressive change in DNA methylation pattern from juvenile to adult individuals (Figure 6b). A dbRDA was conducted using all available variables (age class, season, sex, and year) to check for significant relationships with DNA methylation. The only variation found to significantly explain variance in the model was age class (*p* = .001). The other variable *p* values were season (*p* = .419), sex (*p* = .169), and year (*p* = .542). Age class along with these other variables was deemed to explain 60.7% of variation in the model; however, the adjusted *R* ^2^ for only significant variables (i.e., age class) was 33.3% of variation meaning that 66.7% was unexplained variation.

## 4 DISCUSSION

This study represents the first effort to investigate the relationship between age and global DNA methylation pattern variation in sharks. The obtained results are consistent with the presence of differences in DNA methylation patterns across different age/life stage groups of lemon sharks. Samples were provided in two different batches from different source material (extracted DNA and fin clips in DMSO), leading to a batch effect in data analysis (Figure 1). DNA methylation has been found to be stable at room temperature, and any loss of DNA methylation would be a result from DNA degradation, so it is plausible that there was a slight difference in DNA quality between the batches due to sample holding that is being reflected in the NMDS analysis. Despite this, the sensitivity of the MSAP technique to sample processing observed in the present work constitutes a valuable result of itself and further informs future studies. Additional factors potentially contributing to batch effects were also explored (e.g., sampling seasonality, sex, year sampled); however, their contribution was not significant (Figures S3 and S4; ANOVA *p*‐values .419, .169, and .542). These results also bear interest for studies relying on samples with different origins.

Beyond the observed batch effect, the differences by age group were visible for combined Batch (1 + 2) analysis (*n* = 29; Figure 1) as well as when each batch (Batch 1, *n* = 10, Figure 3; and Batch 2, *n* = 19, Figure 5) was analyzed individually. The consistency of the age group pattern regardless of the batch analyzed further supports a strong link between age and DNA methylation patterns across age groups. To evaluate the observed age group differences, further analyses were conducted using batch 2 samples only in order to remove any interference from the batch effect (Figures 6 and 7) based on the larger sample size of this batch (*n* = 19).

### 4.1 The importance of age clustering for clock development

The findings of this study provide the first evidence for the role of epigenetics during aging in elasmobranchs including sharks, a mysterious group known for the long life spans in some of its members. Previous studies on the whale shark reported that global DNA methylation patterns were similar to other vertebrate species (i.e., high levels of methylation at most CpG sites except for those at promoter regions), leading to the conclusion that this epigenetic modification plays a similar role in sharks as that characterized in other vertebrates (Peat et al., 2017). The present study further supports that conclusion, based on the similarity between the observed age‐related changes in DNA methylation patterns in lemon sharks and other vertebrates (Anastasiadi & Piferrer, 2020; Beal et al., 2019; Bors et al., 2021; De Paoli‐Iseppi et al., 2019; Fei et al., 2021; Polanowski et al., 2014; Stubbs et al., 2017). The largest age‐related change in DNA methylation patterns was found between juveniles and adults seen in the DAPC analysis (Figure 6a), with approximately 50% of the differences between groups explained by discriminant function 1 (x‐axis Figure 6a,b). Interestingly, subadult DNA methylation patterns had a large amount of variation that spanned between the juvenile and adult groups (Figure 6b). These results seem to be in line with reports describing gradual overall changes in DNA methylation throughout the genome correlated with chronological aging (Bollati et al., 2009; Ciccarone et al., 2018; Day et al., 2013; Horvath, 2013; Maegawa et al., 2010). Along the continuum of age, hypomethylation has been observed across the genome due to drift and errors in maintenance of DNA methylation on the genome (Berdyshev et al., 1967; Horvath, 2013; Martin‐Herranz et al., 2019; Shimoda et al., 2014; Wilson et al., 1987). However, this is not the case for all sites as revealed by the presence of some sites across the genome displaying age‐driven hypermethylation (Rakyan et al., 2010). Some of these changes are possibly correlated with changes in gene expression during development (Calvanese et al., 2009; López‐Otín et al., 2013; Pal & Tyler, 2016; Unnikrishnan et al., 2019). This bi‐directional change along with the specificity of individual CpG sites correlating to age could explain why subadults, as observed in this study, have a wider variation in DNA methylation points that seems to span between juveniles and adults as they transition between these two age groups. It is also possible that this same bi‐directional change is one reason that the differentiation between subadults and adults is less obvious and may require more higher resolution methods to elucidate age for these groups.

Another interesting pattern observed in this work is the larger amount of variation in DNA methylation patterns for older shark individuals. Many of these DNA methylation positions most likely are driven by the onset of epigenetic modifications linked to changes in gene expression related to aging; however, many may very well be attributed to the higher incidence of epigenetic drift later in life associated with heterogeneous environments (Jung et al., 2017), contributing to the variation observed, especially since these samples come from varying sample years. This effect is best illustrated by human twin studies revealing a variation in epigenomes driven by environmental heterogeneity (Bell & Spector, 2011; Fraga et al., 2005) but has also been captured by epigenetic clock studies (Beal et al., 2019; Robeck et al., 2021; Stubbs et al., 2017; Thompson et al., 2017).

Although the results reported in this study support a place for MSAP in aging studies, the prevalence of such random and environmentally driven DNA methylation changes during aging prevents this technique from producing accurate estimations of chronological age. On the other hand, MSAP proved useful for making general assignments of age group. Furthermore, these findings suggest that the epigenetic aging pattern observed in mammals also extends to sharks, validating the widespread role of DNA methylation in such process.

### 4.2 The manifold implications of the age of the shark

Future development of epigenetic clocks in sharks would benefit shark conservation similar to the benefits that have been found in being able to characterize the direct link between age, environmental stimulus, and age‐related diseases developed in humans (Bektas et al., 2018; Cavalli & Heard, 2019; Dugué et al., 2018; Zhang et al., 2017). Better understanding of this connection in sharks would allow for environmental threats impacting populations to be identified, as well as the specific age groups most vulnerable to the threats, something that is little known for most shark species. Additionally, with little to no age data existing for most shark populations, little demographic and life history information is available, and the status of populations is hard to assess (Carlson & Goldman, 2007; Goldman & Cailliet, 2004). The current methods used to age sharks (band central staining and bomb radiocarbon aging) are not applicable to all species, require sacrificing individuals, and are fraught with high variation (Campana, 2001; Francis et al., 2007; Huveneers et al., 2013; Natanson et al., 2018). Furthermore, being able to estimate shark age from tissue would greatly enhance the conservation work being done in the fin trade which has been identified as one of the top threats to endangered shark species (Walls & Dulvy, 2020). Current molecular methods only allow for the identification of species and possibly the origin population (Cardeñosa et al., 2020, 2021; Clarke et al., 2004, 2006; Fields et al., 2015, 2018) as well as investigations into the quantities of metals and other pollutants (Barcia et al., 2020; Nalluri et al., 2014) these sharks are exposed to but do not allow for the estimation of age.

Epigenetic alteration is one of the hallmarks of aging, and the study of these alterations could lead to a better understanding and ability to combat age‐related diseases (López‐Otín et al., 2013). As such, studying the specific markers that correlate with age for sharks may prove to be beneficial to understanding and learning how to treat age‐related diseases in humans. Many sharks live long lives (Christiansen et al., 2016; Natanson & Skomal, 2015; Nielsen et al., 2016), and one means to their longevity is thought to be that sharks are less susceptible to age‐related diseases. For example, sharks may be resistant to the age‐related cancers due to potential tumor‐suppressing properties found in their cartilage (although research remains inconclusive) (Oliveira et al., 2019; Walsh et al., 2013); however, there remains a lack of systematic surveys and studies to confirm the actual incidence of cancer in sharks in the first place (Ostrander et al., 2004). Findings from the white shark genome do provide evidence that if resistance to cancer does exist for sharks, it may be due to positive selection of genome stabilizing genes which would have a direct effect upon tumor suppression since many tumors form due to breaks and damage to the DNA (Marra et al., 2019). Further study into epigenetic clocks may provide more insights into the longevity many sharks have and could provide insights into human health and aging.

## 5 CONCLUSIONS

This study contributes to fill two major gaps in the study of shark aging and epigenetics: sharks do exhibit DNA methylation patterns that change with age, and the MSAP method is efficient in identifying such changes. These findings are important because they support the development of epigenetic aging tools in this group of elasmobranchs, fostering the development of additional resources including the generation of full reference genomes. In addition, this work underscores the role of MSAP as a simple, inexpensive, and widely applicable technique to perform the first round of analyses within more elaborated epigenetic pipelines able to systematically estimate chronological age in samples from diverse origins and age groups. Overall, this study provides a first step toward the development of such pipelines for the shark research community with critical implications in conservation and management (Marra et al., 2019).

## AUTHOR CONTRIBUTIONS

**Andria Beal**: Lead Conceptualization, Lead Data curation, Formal analysis, Lead Funding acquisition, Lead Investigation, Methodology, Project administration, Lead Visualization, Lead Writing – original draft. **Serena Hackerott:** Lead Formal analysis, Writing – original draft, Writing – review & editing. **Kevin Feldheim**: Data curation, Funding acquisition, Project administration, Resources, Supervision, Writing – original draft, Writing – review & editing. **Samuel Gruber**: Data curation, Resources. **Jose Maria Eirin‐Lopez**: Conceptualization, Funding acquisition, Project administration, Resources, Supervision, Visualization, Writing – original draft‐, and Writing – review & editing.

## CONFLICT OF INTEREST

All authors claim no conflict of interest.

## Supporting information


Appendix S1

Figures S1–S8

## Data Availability

Data including MSAP datasets with metadata and R code are deposited in the repository of the CREST‐CAChE NSF Center at Florida International University and publicly available at https://github.com/eelabfiu/sharkage.

## References

[ece39226-bib-0001] Anastasiadi, D. , & Piferrer, F. (2020). A clockwork fish: Age prediction using DNA methylation‐based biomarkers in the European seabass. Molecular Ecology Resources, 20, 387–397. 10.1111/1755-0998.13111 31674713

[ece39226-bib-0002] Anderson, M. J. (2001). A new method for non‐parametric multivariate analysis of variance. Austral Ecology, 26(1), 32–46. 10.1111/j.1442-9993.2001.01070.pp.x

[ece39226-bib-0003] Anderson, M. J. , Ellingsen, K. E. , & McArdle, B. H. (2006). Multivariate dispersion as a measure of Beta diversity. Ecology Letters, 9(6), 683–693. 16706913 10.1111/j.1461-0248.2006.00926.x

[ece39226-bib-0004] Barcia, L. G. , Argiro, J. , Babcock, E. A. , Cai, Y. , Shea, S. K. H. , & Chapman, D. D. (2020). Mercury and arsenic in processed fins from nine of the Most traded shark species in the Hong Kong and China dried seafood markets: The potential health risks of shark fin soup. Marine Pollution Bulletin, 157, 111281. 10.1016/j.marpolbul.2020.111281 32469749

[ece39226-bib-0005] Barratclough, A. , Smith, C. R. , Gomez, F. M. , Photopoulou, T. , Takeshita, R. , Pirotta, E. , Thomas, L. , McClain, A. M. , Parry, C. , Zoller, J. A. , Horvath, S. , & Schwacke, L. H. (2021). Accurate epigenetic aging in bottlenose dolphins (*Tursiops truncatus*), an essential step in the conservation of at‐risk dolphins. Journal of Zoological and Botanical Gardens, 2, 416–420. 10.20944/preprints202107.0075.v1

[ece39226-bib-0006] Beal, A. P. , Hackerott, S. , Franks, B. , Gruber, S. H. , Feldheim, K. , & Eirin‐Lopez, J. M. (2021). Epigenetic responses in juvenile lemon sharks (*Negaprion brevirostris*) during a coastal dredging episode in Bimini, Bahamas. Ecological Indicators, 127, 107793. 10.1016/j.ecolind.2021.107793

[ece39226-bib-0007] Beal, A. P. , Kiszka, J. J. , Wells, R. S. , & Eirin‐Lopez, J. M. (2019). The Bottlenose Dolphin Epigenetic Aging Tool (BEAT): A molecular age estimation tool for small cetaceans. Frontiers in Marine Science, 6, 561. 10.3389/fmars.2019.00561

[ece39226-bib-0008] Bektas, A. , Schurman, S. H. , Sen, R. , & Ferrucci, L. (2018). Aging, inflammation and the environment. Experimental Gerontology, 105, 10–18. 10.1016/j.exger.2017.12.015 29275161 PMC5909704

[ece39226-bib-0009] Bell, J. T. , & Spector, T. D. (2011). A twin approach to unraveling epigenetics. Trends in Genetics: TIG, 27(3), 116–125. 21257220 10.1016/j.tig.2010.12.005 PMC3063335

[ece39226-bib-0010] Berdyshev, G. D. , Korotaev, G. K. , Boiarskikh, G. V. , & Vaniushin, B. F. (1967). Nucleotide composition of DNA and RNA from somatic tissues of humpback and its changes during spawning. Biokhimiia, 32(5), 988–993. 5628601

[ece39226-bib-0011] Bollati, V. , Schwartz, J. , Wright, R. , Litonjua, A. , Tarantini, L. , Suh, H. , Sparrow, D. , Vokonas, P. , & Baccarelli, A. (2009). Decline in genomic DNA methylation through aging in a cohort of elderly subjects. Mechanisms of Ageing and Development, 130, 234–239. 10.1016/j.mad.2008.12.003 19150625 PMC2956267

[ece39226-bib-0012] Bors, E. K. , Scott Baker, C. , Wade, P. R. , O'Neill, K. , Shelden, K. E. W. , Thompson, M. J. , Fei, Z. , Jarman, S. , & Horvath, S. (2021). An epigenetic clock to estimate the age of living beluga whales. Evolutionary Applications, 14, 1263–1273. 10.1101/2020.09.28.317610 34025766 PMC8127720

[ece39226-bib-0013] Brooks, J. L. , Guttridge, T. L. , Franks, B. R. , Grubbs, R. D. , Chapman, D. D. , Gruber, S. H. , Dibattista, J. D. , & Feldheim, K. A. (2016). Using genetic inference to re‐evaluate the minimum longevity of the lemon shark *Negaprion brevirostris* . Journal of Fish Biology, 88, 2067–2074. 10.1111/jfb.12943 27060882

[ece39226-bib-0014] Brown, C. A. , & Gruber, S. H. (1988). Age assessment of the lemon shark, *Negaprion brevirostris*, using tetracycline validated vertebral Centra. Copeia, 1988, 747. 10.2307/1445397

[ece39226-bib-0015] Calvanese, V. , Lara, E. , Kahn, A. , & Fraga, M. F. (2009). The role of epigenetics in aging and age‐related diseases. Ageing Research Reviews, 8, 268–276. 10.1016/j.arr.2009.03.004 19716530

[ece39226-bib-0016] Campana, S. E. (2001). Accuracy, precision and quality control in age determination, including a review of the use and abuse of age validation methods. Journal of Fish Biology, 59(2), 197–242. 10.1111/j.1095-8649.2001.tb00127.x

[ece39226-bib-0017] Cardeñosa, D. , Fields, A. T. , Babcock, E. A. , Shea, S. K. H. , Feldheim, K. A. , & Chapman, D. D. (2020). Species composition of the largest shark fin retail‐market in mainland China. Scientific Reports, 10(1), 12914. 32737392 10.1038/s41598-020-69555-1 PMC7395743

[ece39226-bib-0018] Cardeñosa, D. , Fields, A. T. , Babcock, E. , Shea, S. K. H. , Feldheim, K. A. , Kraft, D. W. , Hutchinson, M. , Herrera, M. A. , Caballero, S. , & Chapman, D. D. (2021). Indo‐Pacific origins of silky shark fins in major shark fin Markets highlights supply chains and management bodies key for conservation. Conservation Letters, 14(1), e12780. 10.1111/conl.12780

[ece39226-bib-0019] Carlson, J. K. , & Goldman, K. J. (2007). Special Issue: Age and growth of chondrichthyan fishes: New methods, techniques and analysis. Springer Science & Business Media.

[ece39226-bib-0020] Cavalli, G. , & Heard, E. (2019). Advances in epigenetics link genetics to the environment and disease. Nature, 571(7766), 489–499. 31341302 10.1038/s41586-019-1411-0

[ece39226-bib-0021] Chen, B. H. , Marioni, R. E. , Colicino, E. , Peters, M. J. , Ward‐Caviness, C. K. , Tsai, P.‐C. , Roetker, N. S. , Just, A. C. , Demerath, E. W. , Guan, W. , Bressler, J. , Fornage, M. , Studenski, S. , Vandiver, A. R. , Moore, A. Z. , Tanaka, T. , Kiel, D. P. , Liang, L. , Vokonas, P. , … Horvath, S. (2016). DNA methylation‐based measures of biological age: Meta‐analysis predicting time to death. Aging, 8(9), 1844–1865. 27690265 10.18632/aging.101020 PMC5076441

[ece39226-bib-0022] Christiansen, H. M. , Campana, S. E. , Fisk, A. T. , Cliff, G. , Wintner, S. P. , Dudley, S. F. J. , Kerr, L. A. , & Hussey, N. E. (2016). Using bomb radiocarbon to estimate age and growth of the white shark, *Carcharodon carcharias*, from the southwestern Indian Ocean. Marine Biology, 163, 1–13. 10.1007/s00227-016-2916-9

[ece39226-bib-0023] Ciccarone, F. , Tagliatesta, S. , Caiafa, P. , & Zampieri, M. (2018). DNA methylation dynamics in aging: How far are we from understanding the mechanisms? Mechanisms of Ageing and Development, 174, 3–17. 29268958 10.1016/j.mad.2017.12.002

[ece39226-bib-0024] Clarke, S. C. , McAllister, M. K. , & Michielsens, C. G. J. (2004). Estimates of shark species composition and numbers associated with the shark fin trade based on Hong Kong auction data. Journal of Northwest Atlantic Fishery Science, 35, 453–465. 10.2960/j.v35.m488

[ece39226-bib-0025] Clarke, S. C. , Magnussen, J. E. , Abercrombie, D. L. , Mcallister, M. K. , & Shivji, M. S. (2006). Identification of shark species composition and proportion in the Hong Kong shark fin market based on molecular genetics and trade records. Conservation Biology, 20, 201–211. 10.1111/j.1523-1739.2005.00247.x 16909673

[ece39226-bib-0026] Day, K. , Waite, L. L. , Thalacker‐Mercer, A. , West, A. , Bamman, M. M. , Brooks, J. D. , Myers, R. M. , & Absher, D. (2013). Differential DNA methylation with age displays both common and dynamic features across human tissues that are influenced by CpG landscape. Genome Biology, 14(9), R102. 24034465 10.1186/gb-2013-14-9-r102 PMC4053985

[ece39226-bib-0027] Dugué, P.‐A. , Bassett, J. K. , Joo, J. E. , Baglietto, L. , Jung, C.‐H. , Wong, E. M. , Fiorito, G. , Schmidt, D. , Makalic, E. , Li, S. , Moreno‐Betancur, M. , Buchanan, D. D. , Vineis, P. , English, D. R. , Hopper, J. L. , Severi, G. , Southey, M. C. , Giles, G. G. , & Milne, R. L. (2018). Association of DNA methylation‐based biological age with health risk factors and overall and cause‐specific mortality. American Journal of Epidemiology, 187(3), 529–538. 29020168 10.1093/aje/kwx291

[ece39226-bib-0028] Dulvy, N. K. , Fowler, S. L. , Musick, J. A. , Cavanagh, R. D. , Kyne, P. M. , Harrison, L. R. , Carlson, J. K. , Davidson, L. N. K. , Fordham, S. V. , Francis, M. P. , Pollock, C. M. , Simpfendorfer, C. A. , Burgess, G. H. , Carpenter, K. E. , Compagno, L. J. V. , Ebert, D. A. , Gibson, C. , Heupel, M. R. , Livingstone, S. R. , … White, W. T. (2014). Extinction risk and conservation of the World's sharks and rays. eLife, 3, e00590. 24448405 10.7554/eLife.00590 PMC3897121

[ece39226-bib-0029] Faith, D. P. , Minchin, P. R. , & Belbin, L. (1987). Compositional dissimilarity as a robust measure of ecological distance. Theory and Models in Vegetation Science, 69, 57–68. 10.1007/978-94-009-4061-1_6

[ece39226-bib-0030] Fei, Z. , Raj, K. , Horvath, S. , & Ake, L. (2021). Universal DNA methylation age across mammalian tissues. Innovation in Aging, 5, 410. 10.1093/geroni/igab046.1588

[ece39226-bib-0031] Feldheim, K. A. , Gruber, S. H. , & Ashley, M. V. (2001). Population genetic structure of the lemon shark (*Negaprion brevirostris*) in the Western Atlantic: DNA microsatellite variation. Molecular Ecology, 10, 295–303. 10.1046/j.1365-294x.2001.01182.x 11298946

[ece39226-bib-0101] Feldheim, K. A. , Gruber, S. H. , & Ashley, M. V. (2004). Reconstruction of parental microsatellite genotypes reveals female polyandry and philopatry in the lemon shark, *Negaprion brevirostris* . Evolution, 58(10), 2332–2342. 10.1111/j.0014-3820.2004.tb01607.x 15562694

[ece39226-bib-0032] Feldheim, K. A. , Gruber, S. H. , Dibattista, J. D. , Babcock, E. A. , Kessel, S. T. , Hendry, A. P. , Pikitch, E. K. , Ashley, M. V. , & Chapman, D. D. (2014). Two decades of genetic profiling yields first evidence of Natal philopatry and long‐term Fidelity to parturition sites in sharks. Molecular Ecology, 23(1), 110–117. 24192204 10.1111/mec.12583

[ece39226-bib-0033] Fields, A. T. , Abercrombie, D. L. , Eng, R. , Feldheim, K. , & Chapman, D. D. (2015). A novel mini‐DNA barcoding assay to identify processed fins from internationally protected shark species. PLoS ONE, 10, e0114844. 10.1371/journal.pone.0114844 25646789 PMC4315593

[ece39226-bib-0034] Fields, A. T. , Fischer, G. A. , Shea, S. K. H. , Zhang, H. , Abercrombie, D. L. , Feldheim, K. A. , Babcock, E. A. , & Chapman, D. D. (2018). Species composition of the international shark fin trade assessed through a retail‐market survey in Hong Kong. Conservation Biology, 32(2), 376–389. 10.1111/cobi.13043 29077226

[ece39226-bib-0035] Fraga, M. F. , Ballestar, E. , Paz, M. F. , Ropero, S. , Setien, F. , Ballestar, M. L. , Heine‐Suñer, D. , Cigudosa, J. C. , Urioste, M. , Benitez, J. , Boix‐Chornet, M. , Sanchez‐Aguilera, A. , Ling, C. , Carlsson, E. , Poulsen, P. , Vaag, A. , Stephan, Z. , Spector, T. D. , Wu, Y.‐Z. , … Esteller, M. (2005). Epigenetic differences Arise during the lifetime of monozygotic twins. Proceedings of the National Academy of Sciences of the United States of America, 102(30), 10604–10609. 16009939 10.1073/pnas.0500398102 PMC1174919

[ece39226-bib-0036] Francis, M. P. , Campana, S. E. , & Jones, C. M. (2007). Age under‐estimation in New Zealand porbeagle sharks (*Lamna nasus*): Is there an upper limit to ages that can be determined from shark vertebrae? Marine and Freshwater Research, 58(1), 10–23. 10.1071/mf06069

[ece39226-bib-0037] Gladyshev, V. N. (2016). Aging: Progressive decline in fitness due to the rising Deleteriome adjusted by genetic, environmental, and stochastic processes. Aging Cell, 15, 594–602. 10.1111/acel.12480 27060562 PMC4933668

[ece39226-bib-0038] Goldman, K. , & Cailliet, G. (2004). Age determination and validation in chondrichthyan fishes. In J. C. Carrier , C. A. Simpfendorfer , M. R. Heithaus , K. E. Yopak (Eds.), Book: Sharks and their relatives (pp. 399–447. CRC Press. 10.1201/9780203491317.pt3

[ece39226-bib-0039] Gower, J. C. (1971). A general coefficient of similarity and some of its properties. Biometrics, 27, 857. 10.2307/2528823

[ece39226-bib-0040] Hannum, G. , Guinney, J. , Zhao, L. , Zhang, L. , Hughes, G. , Sadda, S. , Klotzle, B. , Bibikova, M. , Fan, J.‐B. , Gao, Y. , Deconde, R. , Chen, M. , Rajapakse, I. , Friend, S. , Ideker, T. , & Zhang, K. (2013). Genome‐wide methylation profiles reveal quantitative views of human aging rates. Molecular Cell, 49(2), 359–367. 23177740 10.1016/j.molcel.2012.10.016 PMC3780611

[ece39226-bib-0041] Herrera, C. M. , & Bazaga, P. (2010). Epigenetic differentiation and relationship to adaptive genetic divergence in discrete populations of the violet Viola Cazorlensis. The New Phytologist, 187(3), 867–876. 20497347 10.1111/j.1469-8137.2010.03298.x

[ece39226-bib-0042] Heyn, H. , Li, N. , Ferreira, H. J. , Moran, S. , Pisano, D. G. , Gomez, A. , Diez, J. , Sanchez‐Mut, J. V. , Setien, F. , Carmona, F. J. , Puca, A. A. , Sayols, S. , Pujana, M. A. , Serra‐Musach, J. , Iglesias‐Platas, I. , Formiga, F. , Fernandez, A. F. , Fraga, M. F. , Heath, S. C. , … Esteller, M. (2012). Distinct DNA methylomes of newborns and centenarians. Proceedings of the National Academy of Sciences of the United States of America, 109(26), 10522–10527. 22689993 10.1073/pnas.1120658109 PMC3387108

[ece39226-bib-0043] Horvath, S. (2013). DNA methylation age of human tissues and cell types. Genome Biology, 14, R115. 10.1186/gb-2013-14-10-r115 24138928 PMC4015143

[ece39226-bib-0044] Horvath, S. , & Raj, K. (2018). DNA methylation‐based biomarkers and the epigenetic clock theory of ageing. Nature Reviews. Genetics, 19(6), 371–384. 10.1038/s41576-018-0004-3 29643443

[ece39226-bib-0045] Huang, X. , Li, S. , Ni, P. , Gao, Y. , Jiang, B. , Zhou, Z. , & Zhan, A. (2017). Rapid response to changing environments during biological invasions: DNA methylation perspectives. Molecular Ecology, 26(23), 6621–6633. 29057612 10.1111/mec.14382

[ece39226-bib-0046] Huveneers, C. , Stead, J. , Bennett, M. B. , Lee, K. A. , & Harcourt, R. G. (2013). Age and growth determination of three sympatric Wobbegong sharks: How reliable is growth band periodicity in Orectolobidae? Fisheries Research, 147, 413–425. 10.1016/j.fishres.2013.03.014

[ece39226-bib-0047] Jombart, T. , & Ahmed, I. (2011). Adegenet 1.3‐1: New tools for the analysis of genome‐wide SNP data. Bioinformatics, 27(21), 3070–3071. 21926124 10.1093/bioinformatics/btr521 PMC3198581

[ece39226-bib-0048] Jombart, T. , Devillard, S. , & Balloux, F. (2010). Discriminant analysis of principal components: A new method for the analysis of genetically structured populations. BMC Genetics, 11, 94. 20950446 10.1186/1471-2156-11-94 PMC2973851

[ece39226-bib-0049] Jung, S.‐E. , Shin, K.‐J. , & Lee, H. Y. (2017). DNA methylation‐based age prediction from various tissues and body fluids. BMB Reports, 50(11), 546–553. 28946940 10.5483/BMBRep.2017.50.11.175 PMC5720467

[ece39226-bib-0050] Legendre, P. , & Anderson, M. J. (1999). Distance‐based redundancy analysis: Testing multispecies responses in multifactorial ecological experiments. Ecological Monographs, 69, 1–24. 10.1890/0012-9615(1999)069[0001:dbratm]2.0.co;2

[ece39226-bib-0051] Legendre, P. , Oksanen, J. , & ter Braak, C. J. F. (2011). Testing the significance of canonical axes in redundancy analysis. Methods in Ecology and Evolution, 2, 269–277. 10.1111/j.2041-210x.2010.00078.x

[ece39226-bib-0052] López‐Otín, C. , Blasco, M. A. , Partridge, L. , Serrano, M. , & Kroemer, G. (2013). The hallmarks of aging. Cell, 153(6), 1194–1217. 23746838 10.1016/j.cell.2013.05.039 PMC3836174

[ece39226-bib-0053] Maegawa, S. , Hinkal, G. , Kim, H. S. , Shen, L. , Zhang, L. , Zhang, J. , Zhang, N. , Liang, S. , Donehower, L. A. , & Issa, J.‐P. J. (2010). Widespread and tissue specific age‐related DNA methylation changes in mice. Genome Research, 20(3), 332–340. 20107151 10.1101/gr.096826.109 PMC2840983

[ece39226-bib-0054] Marra, N. J. , Stanhope, M. J. , Jue, N. K. , Wang, M. , Sun, Q. , Bitar, P. P. , Richards, V. P. , Komissarov, A. , Rayko, M. , Kliver, S. , Stanhope, B. J. , Winkler, C. , O'Brien, S. J. , Antunes, A. , Jorgensen, S. , & Shivji, M. S. (2019). White shark genome reveals ancient elasmobranch adaptations associated with wound healing and the maintenance of genome stability. Proceedings of the National Academy of Sciences of the United States of America, 116(10), 4446–4455. 30782839 10.1073/pnas.1819778116 PMC6410855

[ece39226-bib-0055] Martin‐Herranz, D. E. , Aref‐Eshghi, E. , Bonder, M. J. , Stubbs, T. M. , Choufani, S. , Weksberg, R. , Stegle, O. , Sadikovic, B. , Reik, W. , & Thornton, J. M. (2019). Screening for genes that accelerate the epigenetic aging clock in humans reveals a role for the H3K36 methyltransferase NSD1. Genome Biology, 20(1), 146. 31409373 10.1186/s13059-019-1753-9 PMC6693144

[ece39226-bib-0056] Mayne, B. , Korbie, D. , Kenchington, L. , Ezzy, B. , Berry, O. , & Jarman, S. (2020). A DNA methylation age predictor for zebrafish. Aging, 12(24), 24817–24835. 33353889 10.18632/aging.202400 PMC7803548

[ece39226-bib-0057] Morán, P. , Marco‐Rius, F. , Megías, M. , Covelo‐Soto, L. , & Pérez‐Figueroa, A. (2013). Environmental induced methylation changes associated with seawater adaptation in Brown trout. Aquaculture, 392–395, 77–83. 10.1016/j.aquaculture.2013.02.006

[ece39226-bib-0058] Morselli, M. , Farrell, C. , Rubbi, L. , Fehling, H. L. , Henkhaus, R. , & Pellegrini, M. (2021). Targeted bisulfite sequencing for biomarker discovery. Methods, 187, 13–27. 32755621 10.1016/j.ymeth.2020.07.006 PMC7855209

[ece39226-bib-0059] Nalluri, D. , Baumann, Z. , Abercrombie, D. L. , Chapman, D. D. , Hammerschmidt, C. R. , & Fisher, N. S. (2014). Methylmercury in dried shark fins and shark fin soup from American restaurants. Science of the Total Environment, 496, 644–648. 10.1016/j.scitotenv.2014.04.107 24835340

[ece39226-bib-0060] Natanson, L. J. , & Skomal, G. B. (2015). Age and growth of the white shark, Carcharodon Carcharias, in the Western North Atlantic Ocean. Marine and Freshwater Research, 66(5), 387–398. 10.1071/mf14127

[ece39226-bib-0061] Natanson, L. J. , Skomal, G. B. , Hoffmann, S. L. , Porter, M. E. , Goldman, K. J. , & Serra, D. (2018). Age and growth of sharks: Do vertebral band pairs record age? Marine and Freshwater Research, 69(9), 1440–1452. 10.1071/mf17279

[ece39226-bib-0062] Nehmens, M. C. , Varney, R. M. , Janosik, A. M. , & Ebert, D. A. (2021). An exploratory study of telomere length in the Deep‐Sea shark, *Etmopterus granulosus* . Frontiers in Marine Science, 8, 642872. 10.3389/fmars.2021.642872

[ece39226-bib-0063] Nielsen, J. , Hedeholm, R. B. , Heinemeier, J. , Bushnell, P. G. , Christiansen, J. S. , Olsen, J. , Ramsey, C. B. , Brill, R. W. , Simon, M. , Steffensen, K. F. , & Steffensen, J. F. (2016). Eye lens radiocarbon reveals centuries of longevity in the Greenland shark (*Somniosus microcephalus*). Science, 353, 702–704. 10.1126/science.aaf1703 27516602

[ece39226-bib-0064] Oksanen, J. , Blanchet, F. G. , Friendly, M. , Kindt, R. , Legendre, P. , McGlinn, D. , Minchin, P. R. , O'Hara, R. B. , Simpson, G. L. , Solymos, P. , Stevens, M. H. , Szoecs, E. , & Wagner, H. (2019). Vegan: Community ecology package. World Agroforestry. https://CRAN.R‐project.org/package=vegan

[ece39226-bib-0065] Oliveira, L. , de Siqueira, L. , de Oliveira, S. , da Silveira Vasconcelos, M. , Mota, E. F. , de Aquino, A. C. , Gomes‐Rochette, N. F. , Nabavi, S. M. , & de Melo, D. F. (2019). Shark Cartilage. In Nonvitamin and nonmineral nutritional supplements, chapter 4.3 (pp. 495–498). Academic Press. 10.1016/b978-0-12-812491-8.00065-5

[ece39226-bib-0066] Ostrander, G. K. , Cheng, K. C. , Wolf, J. C. , & Wolfe, M. J. (2004). Shark cartilage, cancer and the growing threat of pseudoscience. Cancer Research, 64(23), 8485–8491. 15574750 10.1158/0008-5472.CAN-04-2260

[ece39226-bib-0067] Pal, S. , & Tyler, J. K. (2016). Epigenetics and aging. Science Advances, 2(7), e1600584. 27482540 10.1126/sciadv.1600584 PMC4966880

[ece39226-bib-0068] De Paoli‐Iseppi, R. , Deagle, B. E. , Polanowski, A. M. , McMahon, C. R. , Dickinson, J. L. , Hindell, M. A. , & Jarman, S. N. (2019). Age estimation in a long‐lived seabird (*Ardenna tenuirostris*) using DNA methylation‐based biomarkers. Molecular Ecology Resources, 19(2), 411–425. 10.1111/1755-0998.12981 30576072

[ece39226-bib-0069] Peat, J. R. , Ortega‐Recalde, O. , Kardailsky, O. , & Hore, T. A. (2017). The elephant shark methylome reveals conservation of epigenetic regulation across jawed vertebrates. F1000Research, 6, 526. 28580133 10.12688/f1000research.11281.1 PMC5437953

[ece39226-bib-0070] Pérez‐Figueroa, A. (2013). Msap: A tool for the statistical analysis of methylation‐sensitive amplified polymorphism data. Molecular Ecology Resources, 13(3), 522–527. 23311622 10.1111/1755-0998.12064

[ece39226-bib-0071] Pierron, F. , Baillon, L. , Sow, M. , Gotreau, S. , & Gonzalez, P. (2014). Effect of low‐dose cadmium exposure on DNA methylation in the endangered European eel. Environmental Science & Technology, 48(1), 797–803. 24328039 10.1021/es4048347

[ece39226-bib-0072] Polanowski, A. M. , Robbins, J. , Chandler, D. , & Jarman, S. N. (2014). Epigenetic estimation of age in humpback whales. Molecular Ecology Resources, 14(5), 976–987. 24606053 10.1111/1755-0998.12247 PMC4314680

[ece39226-bib-0074] Rakyan, V. K. , Down, T. A. , Maslau, S. , Andrew, T. , Yang, T.‐P. , Beyan, H. , Whittaker, P. , McCann, O. T. , Finer, S. , Valdes, A. M. , Leslie, R. D. , Deloukas, P. , & Spector, T. D. (2010). Human aging‐associated DNA hypermethylation occurs preferentially at bivalent chromatin domains. Genome Research, 20(4), 434–439. 20219945 10.1101/gr.103101.109 PMC2847746

[ece39226-bib-0075] Remot, F. , Ronget, V. , Froy, H. , Rey, B. , Gaillard, J.‐M. , Nussey, D. H. , & Lemaître, J.‐F. (2020). No sex differences in adult telomere length across vertebrates: A meta‐analysis. Royal Society Open Science, 7(11), 200548. 33391781 10.1098/rsos.200548 PMC7735339

[ece39226-bib-0076] Reyna‐López, G. E. , Simpson, J. , & Ruiz‐Herrera, J. (1997). Differences in DNA methylation patterns are detectable during the dimorphic transition of fungi by amplification of restriction polymorphisms. Molecular & General Genetics: MGG, 253(6), 703–710. 9079881 10.1007/s004380050374

[ece39226-bib-0077] Robeck, T. R. , Fei, Z. , Lu, A. T. , Haghani, A. , Jourdain, E. , Zoller, J. A. , Li, C. Z. , Steinman, K. J. , DiRocco, S. , Schmitt, T. , Osborn, S. , van Bonn, B. , Katsumata, E. , Mergl, J. , Almunia, J. , Rodriguez, M. , Haulena, M. , Dold, C. , & Horvath, S. (2021). Multi‐species and multi‐tissue methylation clocks for age estimation in toothed whales and dolphins. Communications Biology, 4(1), 642. 34059764 10.1038/s42003-021-02179-x PMC8167141

[ece39226-bib-0078] Rodríguez‐Casariego, J. A. , Mercado‐Molina, A. E. , Garcia‐Souto, D. , Ortiz‐Rivera, I. M. , Lopes, C. , Baums, I. B. , Sabat, A. M. , & Eirin‐Lopez, J. M. (2020). Genome‐wide DNA methylation analysis reveals a conserved epigenetic response to seasonal environmental variation in the staghorn coral Acropora Cervicornis. Frontiers in Marine Science, 7, 822.

[ece39226-bib-0079] Salameh, Y. , Bejaoui, Y. , & El Hajj, N. (2020). DNA methylation biomarkers in aging and age‐related diseases. Frontiers in Genetics, 11, 171. 10.3389/fgene.2020.00171 32211026 PMC7076122

[ece39226-bib-0080] Shimoda, N. , Izawa, T. , Yoshizawa, A. , Yokoi, H. , Kikuchi, Y. , & Hashimoto, N. (2014). Decrease in cytosine methylation at CpG Island shores and increase in DNA fragmentation during zebrafish aging. AGE, 36, 103–115. 10.1007/s11357-013-9548-5 23736955 PMC3889898

[ece39226-bib-0081] Stubbs, T. M. , Team, B. I. A. C. , Bonder, M. J. , Stark, A.‐K. , Krueger, F. , von Meyenn, F. , Stegle, O. , & Reik, W. (2017). Multi‐tissue DNA methylation age predictor in mouse. Genome Biology, 18, 68. 10.1186/s13059-017-1203-5 28399939 PMC5389178

[ece39226-bib-0082] Suarez‐Ulloa, V. , Rivera‐Casas, C. , & Michel, M. (2019). Seasonal DNA methylation variation in the flat tree oyster Isognomon Alatus from a mangrove ecosystem in north Biscayne Bay, Florida. Journal of Shellfish Research, 38(1), 79–88. 10.2983/035.038.0108

[ece39226-bib-0083] Sunnucks, P. , & Hales, D. F. (1996). Numerous transposed sequences of mitochondrial cytochrome oxidase I‐II in aphids of the genus Sitobion (Hemiptera: Aphididae). Molecular Biology and Evolution, 13(3), 510–524. 8742640 10.1093/oxfordjournals.molbev.a025612

[ece39226-bib-0084] Tanabe, A. , Shimizu, R. , Osawa, Y. , Suzuki, M. , Ito, S. , Goto, M. , Pastene, L. A. , Fujise, Y. , & Sahara, H. (2020). Age estimation by DNA methylation in the Antarctic Minke whale. Fisheries Science: FS, 86(1), 35–41.

[ece39226-bib-0085] Thompson, M. J. , von Holdt, B. , Horvath, S. , & Pellegrini, M. (2017). An epigenetic aging clock for dogs and wolves. Aging, 9, 1055–1068. 10.18632/aging.101211 28373601 PMC5391218

[ece39226-bib-0086] Trigg, S. A. , Venkataraman, Y. R. , Gavery, M. R. , Roberts, S. B. , Bhattacharya, D. , Downey‐Wall, A. , Eirin‐Lopez, J. M. , Johnson, K. M. , Lotterhos, K. E. , Puritz, J. B. , & Putnam, H. M. (2021). Invertebrate methylomes provide insight into mechanisms of environmental tolerance and reveal methodological biases. Molecular Ecology Resources, 22, 1247–1261. 10.1111/1755-0998.13542 34709728

[ece39226-bib-0087] Unnikrishnan, A. , Freeman, W. M. , Jackson, J. , Wren, J. D. , Porter, H. , & Richardson, A. (2019). The role of DNA methylation in epigenetics of aging. Pharmacology & Therapeutics, 195, 172–185. 10.1016/j.pharmthera.2018.11.001 30419258 PMC6397707

[ece39226-bib-0088] Vaiserman, A. , & Krasnienkov, D. (2020). Telomere length as a marker of biological age: State‐of‐the‐art, open issues, and future perspectives. Frontiers in Genetics, 11, 630186. 33552142 10.3389/fgene.2020.630186 PMC7859450

[ece39226-bib-0090] Walls, R. H. L. , & Dulvy, N. K. (2020). Eliminating the dark matter of data deficiency by predicting the conservation status of Northeast Atlantic and Mediterranean Sea sharks and rays. Biological Conservation, 246, 108459. 10.1016/j.biocon.2020.108459

[ece39226-bib-0091] Walsh, C. , Luer, C. , Yordy, J. , Cantu, T. , Miedema, J. , Leggett, S. , Leigh, B. , Adams, P. , Ciesla, M. , Bennett, C. , & Bodine, A. B. (2013). Epigonal conditioned media from Bonnethead shark, Sphyrna Tiburo, induces apoptosis in a T‐cell leukemia cell line, Jurkat E6‐1. Marine Drugs, 11, 3224–3257. 10.3390/md11093224 24065163 PMC3806469

[ece39226-bib-0092] Wilson, V. L. , Smith, R. A. , Ma, S. , & Cutler, R. G. (1987). Genomic 5‐Methyldeoxycytidine decreases with age. The Journal of Biological Chemistry, 262(21), 9948–9951. 3611071

[ece39226-bib-0093] Zhang, Y. , Wilson, R. , Heiss, J. , Breitling, L. P. , Saum, K.‐U. , Schöttker, B. , Holleczek, B. , Waldenberger, M. , Peters, A. , & Brenner, H. (2017). DNA methylation signatures in peripheral blood strongly predict all‐cause mortality. Nature Communications, 8, 14617. 10.1038/ncomms14617 PMC5357865 28303888

[ece39226-bib-0094] Zhao, Y. , Chen, M. , Storey, K. B. , Sun, L. , & Yang, H. (2015). DNA methylation levels analysis in four tissues of sea cucumber *Apostichopus japonicus* based on fluorescence‐labeled methylation‐sensitive amplified polymorphism (F‐MSAP) during aestivation. Comparative Biochemistry and Physiology. Part B, Biochemistry & Molecular Biology, 181, 26–32. 10.1016/j.cbpb.2014.11.001 25461675

