# Supplementary material for: Age group DNA methylation differences in lemon sharks (Negaprion brevirostris): Implications for future age estimation tools
Source: Ecol Evol. 2022 Aug 29;12(8):e9226. doi: 10.1002/ece3.9226 (PMC9425014; doi:10.1002/ece3.9226)
Supplement: Supplementary file 2 — Figures S1–S8 [file ECE3-12-e9226-s002.docx]

Supplementary Figures for Beal et al.

**Preliminary Analysis with Batch 1 samples**

The condition for -/- was set to uninformative rather than being assumed full methylation (as in the rest of the manuscript) for this analysis. A total of 308 loci were identified by the two primers (primer A: 120 loci and primer B: 106 loci), with 262 loci identified as Methylation-Susceptible Loci (MSL), and 32 loci identified as Non-Methylated Loci (NML). The three different age classes, Adult, Subadult, and Young of Year (Juvenile), showed slightly different overall patterns of the four types of DNA methylation identified through the MSAP analysis.


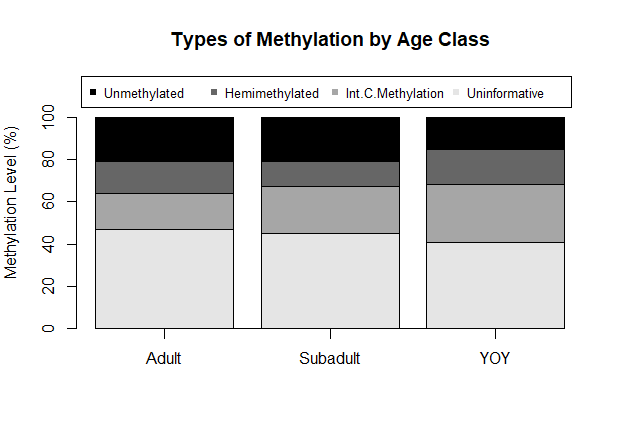


**Figure S1-Levels of the Types of Methylation for each Age Class for Preliminary Analysis of Shark Age Groups**. The level of methylation (%) at MSL for each of the four types of DNA Methylation (Unmethylated, Hemi-methylated, Methylated at an Internal Cytosine, and Uninformative) for each age class.


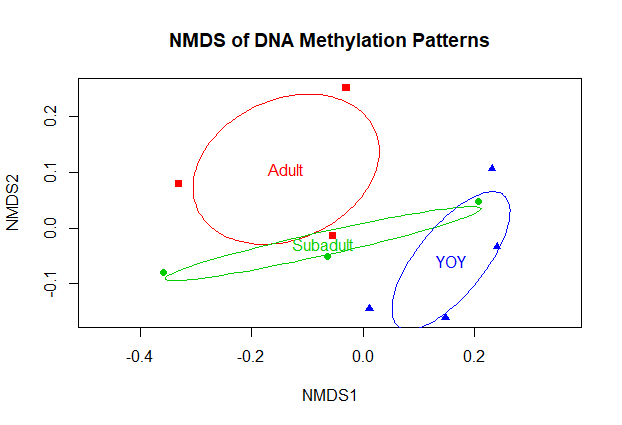


**Figure S2-Nonmetric Multidimensional Scaling (NMDS) Plot for Preliminary Analysis of Shark Age Groups**. Plot of NMDS analysis of MSL (Stress: 0.0730) with samples from the Adult age class shown in red, from the Subadult age class shown in green, and from the Young of Year (YOY) shown in blue. Ellipses represent standard deviation of points.

**Combined Samples**


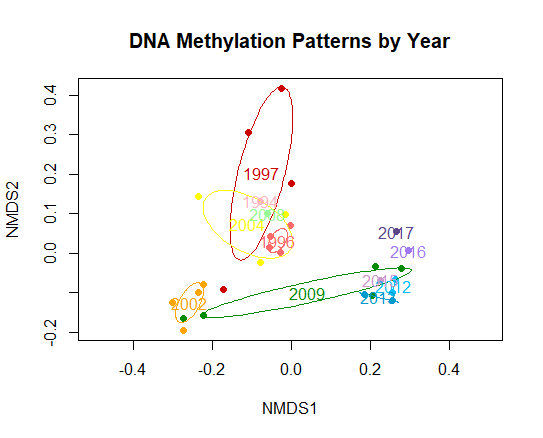


**Figure S3- NMDS plot for combined samples by year of sampling.** Individual data points color coded by year sampled and ellipses represent standard deviation of points.


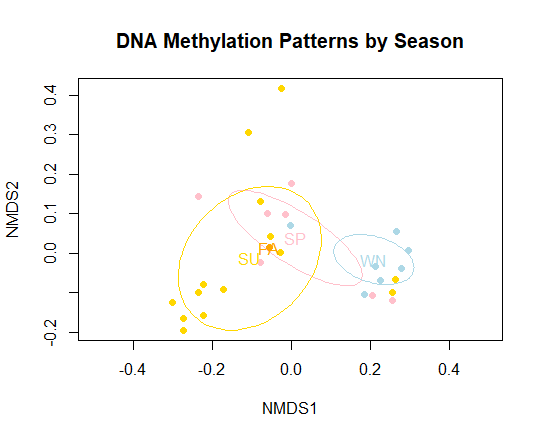


**Figure S4- NMDS plot for combined batches by season.** Individual data points colored by season (Summer- Gold, Fall- Orange, Winter- Blue, and Spring- Pink).

**BATCH 1 MSAP Package plot and NMDS**


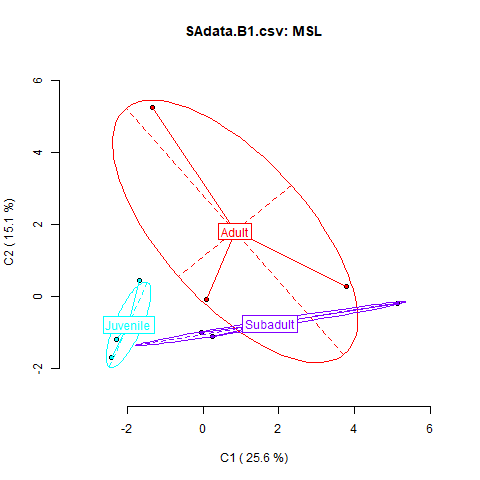


**Figure S5- MSAP Package PCoA for batch 1. Each dot represents an individual and it’s distance from another dot represents the difference/similarity of the two individuals DNA methylation pattern.**


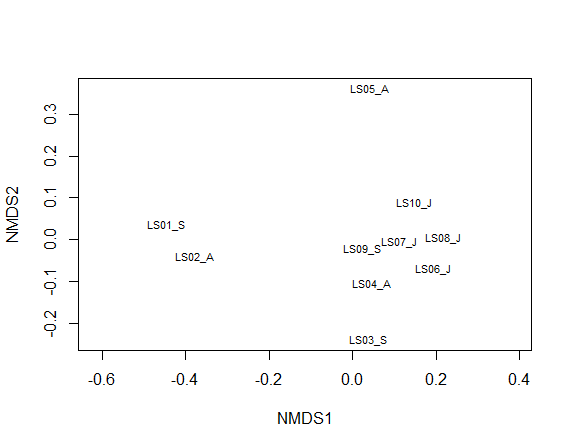


**Figure S6- NMDS plot for batch 1 with sample ID. Distance between any two individuals represents the difference/similarity of DNA methylation pattern between those two individuals.**

**BATCH 2 MSAP Package Plot and NMDS**


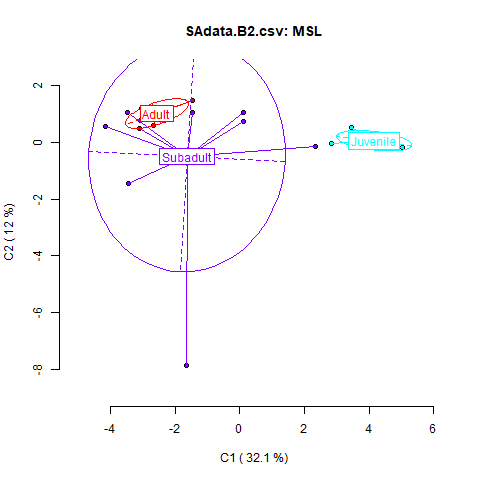


**Figure S7- MSAP Package PCoA for Batch 2. Each dot represents an individual and it’s distance from another dot represents the difference/similarity of the two individuals DNA methylation pattern.**


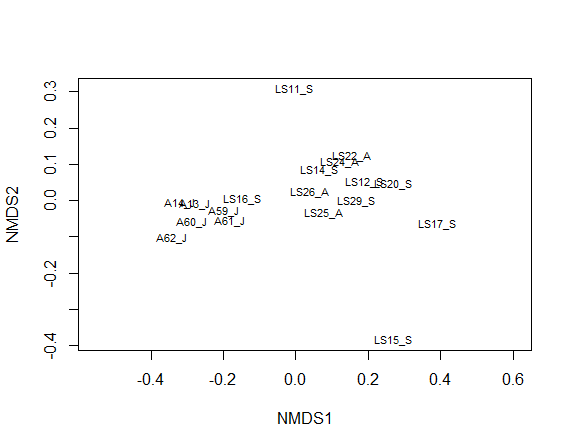


**Figure S8- NMDS plot for batch 2 with sample IDs. Distance between any two individuals represents the difference/similarity of DNA methylation pattern between those two individuals.**
